# Chemical Looping with Oxygen Uncoupling of Biomass and Coal: Scaling-Up to 50 kW_th_ Using a Copper-Based Oxygen Carrier

**DOI:** 10.1021/acs.energyfuels.4c03173

**Published:** 2024-10-28

**Authors:** A. Abad, A. Filsouf, I. Adánez-Rubio, T. Mendiara, L. F. de Diego, M. T. Izquierdo, P. Gayán, J. Adánez

**Affiliations:** Department of Energy and Environment, 120031 Instituto de Carboquímica (ICB), CSIC, 50018 Zaragoza, Spain

## Abstract

Chemical looping with oxygen uncoupling (CLOU) is an advanced CO_2_ capture technology where an oxygen carrier supplies the gaseous oxygen required for combustion. The technology has experienced a significant development degree in the most recent decades, up to TRL 4. This study presents results that further prove the viability of this technology to TRL 5 in a continuous CLC unit with two interconnected circulating fluidized beds acting as fuel and air reactors. The oxygen carrier used in the experiments was a magnetic copper-based oxygen carrier denoted as Cu30MnFe. It was used in the combustion of three types of biomass and a bituminous coal (Taldinsky coal). The effects on char conversion, CO_2_ capture efficiency, and total oxygen demand of different parameters such as excess air ratio, fuel reactor temperature, residence time in fuel reactor, gas velocity in the carbon stripper, solid circulation rate, and solid inventory in fuel reactor were investigated. CO_2_ capture efficiency of 93% was achieved at 886 °C with pine sawdust combustion, while the total oxygen demand was 4.6%. In the experiments with bituminous coal, the effectiveness of the carbon stripper in improving the CO_2_ capture efficiency was demonstrated. During the highest load of coal, low total oxygen demands were achieved even at temperatures below 900 °C.

## Graphical abstract

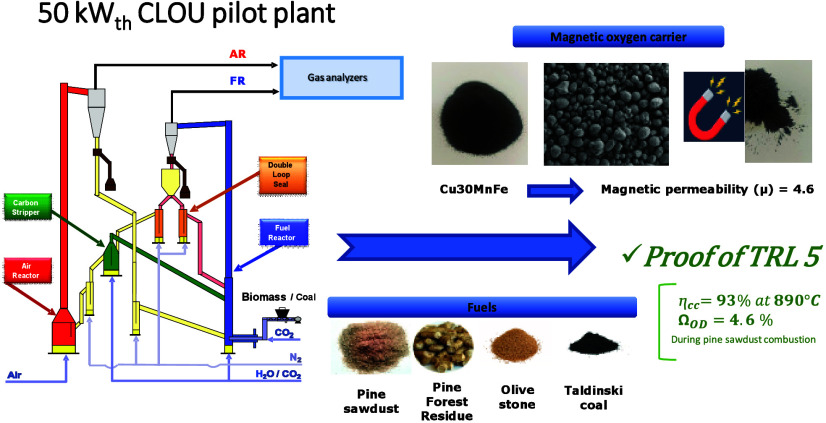

## 1 Introduction

The chemical looping combustion (CLC) technique is well-suited for capturing carbon dioxide emissions during combustion, thereby mitigating its impact on climate change. It holds the potential to significantly lower the costs associated with carbon dioxide capture.^1^ Chemical looping with oxygen uncoupling (CLOU) represents a promising advancement from CLC that facilitates the combustion of solid fuels. The CLOU process operates with oxygen carriers that liberate gaseous oxygen within the fuel reactor, improving combustion of the solid fuel with oxygen in the gas phase. This approach avoids the slow gasification step of the char and significantly accelerates solid conversion rates.^2^

Figure 1 illustrates the scheme of a CLOU unit. In the fuel reactor (FR), the oxygen carrier undergoes decomposition to generate gaseous oxygen (reaction [Disp-formula eqr1]). The fuel is devolatilized into volatiles and char (reaction [Disp-formula eqr2]). Subsequently, the volatiles and char interact with the liberated oxygen in the fuel reactor according to reactions [Disp-formula eqr3] and [Disp-formula eqr4]. The reduced oxygen carrier is conveyed to the air reactor (AR) for reoxidation reaction [Disp-formula eqr5], after which it is transferred back to the fuel reactor to release gaseous oxygen. If the char does not completely react with oxygen in the fuel reactor, a carbon stripper unit operating between fuel and air reactors would facilitate the separation of the char and its return to the fuel reactor to complete reaction [Disp-formula eqr4].^3^ In any case, to avoid the accumulation of fuel ashes in the continuous unit, it would be necessary to periodically drain the ashes from the system.

$$2{\mathrm{Me}}_{x}O_{y}↔2{\mathrm{Me}}_{x}O_{y-1}+O_{2}$$ r1

$$\mathrm{fuel}\to \mathrm{char}+\mathrm{volatiles}+H_{2}O$$ r2

$$\mathrm{volatile}+O_{2}\to {\mathrm{CO}}_{2}+H_{2}O$$ r3

$$\mathrm{char}+O_{2}\to {\mathrm{CO}}_{2}$$ r4

$$2{\mathrm{Me}}_{x}O_{y-1}+O_{2}↔2{\mathrm{Me}}_{x}O_{y}$$ r5

**Figure 1:**
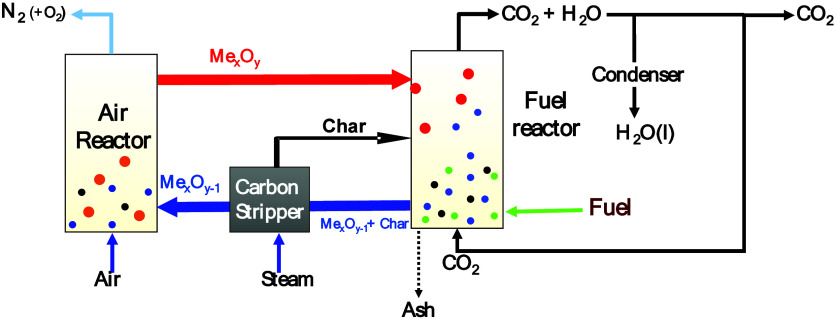
Scheme of a CLOU unit.

Three metal oxide systems with favorable thermodynamic properties have been identified for the CLOU process: CuO/Cu_2_O, Mn_2_O_3_/Mn_3_O_4_, Co_3_O_4_/CoO, and their mixed oxides.^4^ Co-based materials have been disregarded considering their high toxicity and cost as well as their availability. Thermodynamic and kinetic limitations on the oxygen uncoupling capability of Mn-based materials make most of the development studies focus on copper materials or at most mixed copper–manganese materials. Most of the studies were conducted at a thermobalance or batch fluidized bed scale. This would correspond to a TRL 2–3 on the technological readiness level scale, which classifies the maturity of a technology from 1 (basic principles) to 9 (commercial). In the most recent years several literature reviews on CLOU materials have been published,^4,5^ focused on Cu-based and Cu mixed oxides oxygen carriers^6^ and their properties. Although some of them were promising, only a limited number of materials were tested in a continuous unit at the laboratory scale, which would correspond to TRL 4. Table 1 summarizes the state of the development and scale-up of the CLOU technology.

**Table 1:** Summary of State-of-the-Art Developments in CLOU Technology

TRL	Research group	Type of oxygen carrier	Degree of development
2–3	University of Utah (USA)^7^	CuO supported on SiO_2_, TiO_2_, ZrO_2_	TGA/batch fluidized bed
	ICB-CSIC (Spain)^2,8^	CuO supported on Al_2_O_3_, ZrO_2_, MgAl_2_O_4_, TiO_2_, SiO_2_	TGA/batch fluidized bed
		CuMnFe, CuMnFek	
	Chalmers University of Technology (Sweden)^3,5^	CuO supported on Al_2_O_3_, ZrO_2_, MgAl_2_O_4_, CeO_2_,	TGA/batch fluidized bed
		CuO/Fe_2_O_3_/MgO, CuO/Fe_2_O_3_/Al_2_O_3_	
		CaMn_*x*_Ti_1–*x*_O_3_	
	University of Cambridge^9^	CuO/Al_2_O_3_	Batch fluidized bed
4	ICB-CSIC (Spain)^10–16^	CuO/MgAl_2_O_4_, CuFe, CuMn, CuMnFe, CuMnFek	1.5 kW_th_ continuous unit
	Chalmers University of Technology (Sweden)^17^	MnSiTi	10 kW_th_ continuous unit
5	Vienna University of Technology (Austria)^18^	C28 (CaTi_0.1_Mn_0.9_O_2.96_)	80 kW_th_ continuous unit
	University of Utah (USA)^19^	20% Cu-on-SiC	200 kW_th_ continuous unit
	ICB-CSIC (Spain)	CuMnFe	50 kW_th_ (this work)

The CLOU process was demonstrated at TRL 4 with a Cu-based oxygen carrier (60 wt % CuO/MgAl_2_O_4_) in a 1.5 kW_th_ CLOU unit at ICB-CSIC (Spain) with both coal^10^ and pine sawdust biomass.^11^ Complete combustion of coal and biomass up to 900 °C was achieved, reaching 100% of CO_2_ capture at 935 °C with pine sawdust. Similar experimental campaigns were conducted in the same unit with mixed oxides as CuFe^12^ and CuMn.^13,14^ Also, magnetic Cu-based oxygen carriers were developed, where the magnetism is given by a MnFe spinel used as support.^15^ Complete combustion of the fuels and high CO_2_ capture efficiencies were achieved regardless of coal type and biomass (except swine manure)^16^ even at the low temperature of 775 °C. These CLOU oxygen carriers did not show any defluidization or agglomeration problems. Moreover, those with magnetic properties maintained them with 99.3% oxygen carrier recovery from fuel ashes. Thus, the loss of oxygen carrier during ash drainage would be avoided, and the oxygen carrier reused.^15^ Also at TRL 4, manganese, silicon, and titanium oxides were tested at Chalmers University of Technology as CLOU oxygen carriers in a 10 kW_th_ plant,^17^ with a power input between 2 and 6 kW_th_. Based on the results, the highest CO_2_ capture efficiency was 99.6% for combustion of lignite, although the oxygen demand was 20%. They did not observe any defluidization or agglomeration of the material during the tests.

During the past decade, there has been an increasing interest in the scale-up of the CLOU process from the previous TRL 4 to TRL 5. One of the cornerstones in this step was the design of the CLC unit itself. The TRL 4 units were based on interconnected bubbling fluidized beds acting as fuel and air reactors, respectively. However, numerical simulations conducted at the University of Utah (USA) showed that the use of circulating fluidized beds (CFBs) for fuel and air reactors allowed better control of the operational parameters than the use of bubbling fluidized beds and also made the scalability to industrial scale easier.^20^ Thus, some of the larger CLOU units to date were constructed under this design. An example is the 80 kW_th_ pilot plant at the Vienna University of Technology for the combustion of solid fuels. In this case, internals in the upper part of the FR were also incorporated, which improved the contact of the unburnt gases from the FR bed with the regenerated oxygen carrier, returned back from AR, just in the middle of the column. In this unit, Fleiβ et al. used a perovskite oxygen carrier (C28) for the combustion of two different solid fuels (softwood and bark) during 16 h of operation.^18^ This oxygen carrier has a low CLOU oxygen transport capacity (0.8 wt % perovskite CaTi_0.1_Mn_0.9_O_2.96_), although, during the experimental campaign, they obtained a very high efficiency of combustion of 99.6% and even a CO_2_ capture efficiency of 98.3% operating in a power interval between 40 and 55 kW_th_. Some issues with the regeneration of the oxygen carrier in the air reactor to recover the CLOU properties were observed due to the formation of non-CLOU phases as CaMn_2_O_4_ and Ca_2_MnO_4_.^18^

A 200 kW_th_ chemical looping unit was erected at the University of Utah (USA) known as process development unit (PDU).^19^ In order to better control the flow between reactors, the PDU was designed so that all of the material recovered by the FR cyclone is recirculated to the same reactor, while all of the material recovered by the AR cyclone is transferred to the FR.^19^ Initial experimental campaigns were carried out with ilmenite as oxygen carrier.^21^ To ensure autothermal operation they have to add propane or natural gas to the air reactor and also enrich with pure oxygen the air in the AR, to be able to operate with a continuous fuel feeding.^21,22^ They changed to a mixture of 12% CLOU Cu-based oxygen carrier (20% Cu-on-SiC) and ilmenite in the bed during 90 min of fuel feeding. The addition of the CLOU oxygen carrier avoided the use of gaseous fuel to maintain the temperature of the system. Thus, although results were promising,^22^ it is necessary to have future experimental campaigns with only CLOU oxygen carrier in the unit.

Also in the frame of TRL 5, the Instituto de Carboquímica (ICB-CSIC) recently constructed and operated a CLC unit where fuel and air reactors are also two interconnected circulating fluidized beds, including a carbon stripper reactor between them to increase the CO_2_ capture rates.^23^ In this unit, different mineral-based oxygen carriers were tested in the combustion of coal^24,25^ and several types of biomass.^26^ In the case of biomass, oxygen demand values in the 10–15% interval were reached, while CO_2_ capture values were close to 95% for the most reactive type of biomass. The design of this unit was based on a thermal power of 20 kW_th_ for chemical looping combustion or 50 kW_th_ for chemical looping with oxygen uncoupling (CLOU).

Thus, based on the experience gained in the operation of this unit as well as the good performance of the CuMnFe CLOU oxygen carrier previously found in a TRL 4 continuous unit, the present work aims to demonstrate for the first time the continuous operation at TRL 5 of a copper-based CLOU oxygen carrier (Cu30MnFe) with different types of fuels. The novelty of this work lies both in the properties of the oxygen carrier used compared to those used until now and in the experimental campaign carried out with it, which includes the systematic analysis of different operating conditions that allow demonstrating the viability of the CLOU process at TRL 5. Relevant information is expected to be obtained for critical issues that must be considered for the future scale-up of CLOU for solid fuel combustion.

## 2 Materials and Methods

### 2.1 Oxygen Carrier Preparation

The oxygen carrier used in this work was synthesized from raw materials including 30 wt % CuO (Panreac), 34 wt % Mn_3_O_4_ (Micromax, Elkem), and 36 wt % Fe_2_O_3_ (Acros Organics). A spouted fluidized bed spray granulator (Glatt W51530 + OPP1) was used to produce 120 kg of particles in the desired range of 100–300 μm. Different additives such as dispersant, deflocculant, and PEO-1 (Sumitomo) were also added in the granulation process. Granulated particles were calcined in air in a muffle at 1050 °C for 8 h. This oxygen carrier was denoted as Cu30MnFe. The main properties of the prepared oxygen carrier are shown in Table 2.

**Table 2:** Main Properties of the Oxygen Carrier, Cu30MnFe

	Fresh
CuO content (wt %)	30
Oxygen transport capacity, *R*_oc_ (wt %)	2.4
Crushing strength (N)	2.8
Magnetic permeability, μ	4.7
Skeletal density of particle (kg/m^3^)	5160
Porosity (%)	6.9
AJI (%)	0.4

### 2.2 Fuels

Three different types of biomass were used as fuels in the experiments. Pine sawdust () with the particle size in the +0.5–3.16 mm interval, pine forest residue (PFR) in the form of pellets with average length of 15 mm and 6 mm diameter, and olive stones (*Olea europaea*) with particle size in the +0.5–2 mm interval. In addition, a medium-volatile bituminous Russian coal, denoted as Taldinsky MVB, with the particle size being +0.1–0.5 mm, was also used as a fuel. Table 3 shows the properties of the different types of biomass and coal used as fuels.

**Table 3:** Characterization of the Biomass and Coal Used As Fuels (Air Dry Basis)

	Pine sawdust	PFR	Olive stones	Taldinsky bituminous coal
Proximate analysis (wt %)				
Moisture	6.9	3.3	9.4	5.8
Volatile matter	73.6	77.2	72.5	32.0
Fixed carbon	17.9	18.3	17.3	52.1
Ash	1.6	1.3	0.8	10.1
Ultimate analysis (wt %)				
C	47.5	51.5	46.5	65.8
H^a^	5.5	5.8	4.8	4.6
N	0.3	0.3	0.2	2.0
S	0.0	0.0	0.0	0.5
O	38.2	37.8	38.3	11.3
LHV (kJ/kg)	19158	17941	16807	26600
*ω*_sf_ (kg of oxygen/(kg of fuel))	1.33	1.46	1.24	2.04

a Free of moisture.

### 2.3 Experimental Setup

Experiments were performed in a CLC unit designed with circulating fluidized beds as fuel and air reactors for 50 kW_th_ solid fuel feeding. Also, a carbon stripper (CS) is included in order to minimize the flow of char entering to the AR. The unit has been described in detail elsewhere, and only a brief description will be included next.^23^ Figure 2 depicts the layout of the plant built at the Instituto de Carboquímica (ICB-CSIC).

**Figure 2:**
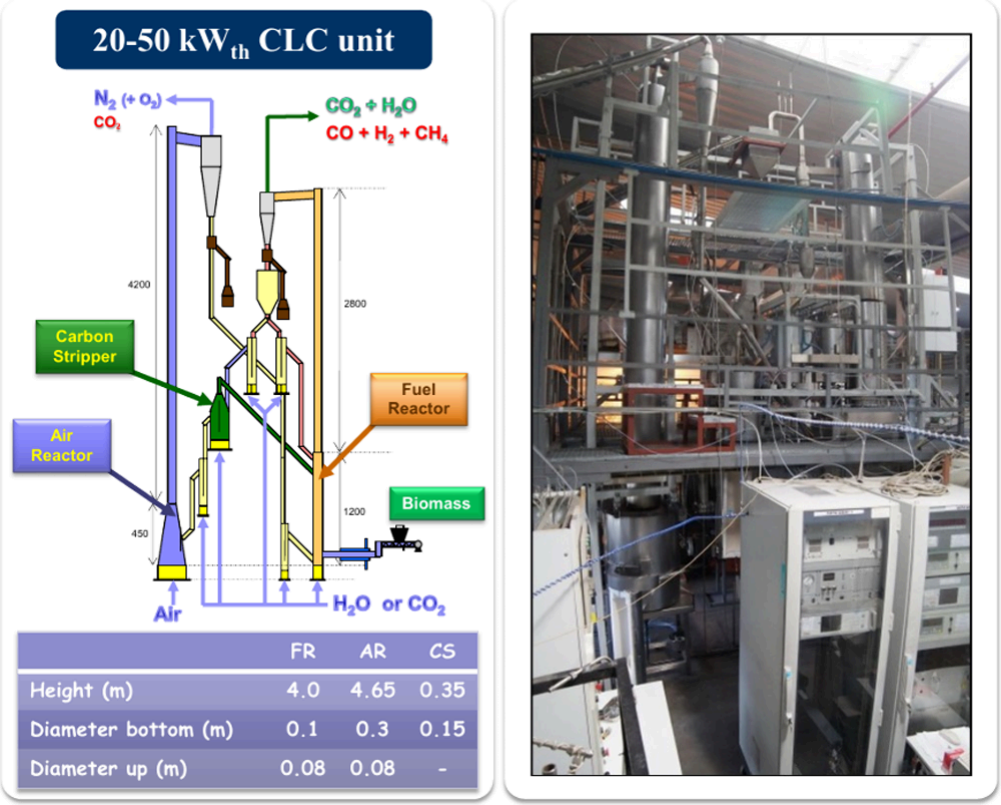
Layout and picture of the 50 kW_th_ CLC unit (ICB-CSIC-s50) for solid fuels.

The main parts of this CLC unit are the two interconnected circulating fluidized beds, the air and fuel reactors, connected with a carbon stripper via loop seal reactors that avoid mixing atmospheres. The fuel reactor is working in the turbulent fluidizing regime. Fuel is fed at the bottom of the fuel reactor through a double screw feeder system to maximize the contact between volatiles and the oxygen carrier. In order to recover the particles escaping from the cyclone at the fuel reactor outlet, a gravitational settling chamber is placed downstream. After the fuel reactor cyclone, there is a double loop seal. The outlet stream is divided in two streams: a portion of solid goes to the carbon stripper, and the rest can be recirculated into the fuel reactor. The solid circulation rate between fuel and air reactors can be controlled by the gas velocity in the double loop seal.^23^ The carbon stripper in the unit was designed as a bubbling fluidized bed. The carbon stripper allowed routing back the elutriated char particles to the fuel reactor to increase the CO_2_ capture of the unit. Air reactor consists of two sections, a wide bottom bed followed by a narrow riser to facilitate the solid circulation rate.

Another important feature of this unit is the possibility to directly measure the solid flux exiting from both the fuel and air reactors by means of diverting solid valves placed below cyclones at the outlet of each reactor. It should be mentioned that the air reactor, the carbon stripper, and the double loop seal in this unit are heated electrically. In order to analyze the gas composition at the outlet of the reactors, an online gas analyzer is used. Infrared analyzers were used to measure methane, carbon monoxide, and carbon dioxide. Hydrogen concentration was determined by a thermal conductivity analyzer and oxygen concentration in a paramagnetic analyzer.

### 2.4 Experimental Conditions

The experimental campaign accounted for 40 h of fuel combustion with the different solid fuels described above. The total solid inventory of Cu30MnFe introduced to the unit was 70 kg. As has been mentioned before, the objective of the experimental campaign was to demonstrate the viability of the CLOU process at a level of TRL 5. Therefore, the analysis of the effect on the behavior of the process of different operating variables was accomplished, such as the air supplied in the air reactor, the fuel reactor temperature (*T*_FR_), the residence time of the solids in the fuel reactor (τ_OC_), the solids circulation rate between reactors (ṁ_OC_), the fluidization velocity in the carbon stripper (*u*_g,in CS_), the fuel feed rate (˙ṁ_FR_), and the solid inventory in the fuel reactor (*m*_FR_). Tables 4 and 5 summarize the variables modified in different experimental series and the corresponding evaluating parameters, i.e., CO_2_ capture efficiency (η_CC_), char conversion (*X*_c_), and total oxygen demand (Ω_OD_).

**Table 4:** Operating Conditions and Main Results in the CLOU Unit during Biomass Combustion^a^

Fuel	Series	Test	*T*_FR_ (°C)	*T*_AR_ (°C)	*ṁ*_OC_ (kg/h)	*ṁ*_SF_ (kg/h)	*m*_FR_ (kg/MW_th_)	τ_OC_ (s)	ϕ (-)	λ (-)	Power (kW_th_)	*T*_CS_ (°C)	*u*_g,in cs_ (m/s)	η_CC_ (%)	*X*_C_ (%)	Ω_OD_ (%)
Pine sawdust	Series I	1	**839**	879	223	1.7	254	35	3.0	**1.9**	8.4	831	0.16	90	73.46	7.4
		2	**858**	897	223	2.0	211	34	2.6	**1.6**	10	823	0.16	89.3	71.57	9.3
	Series II	3	**784**	809	182	2.2	189	41	1.9	3.5	11	794	0.13	88.1	68.5	4.7
		4	**774**	804	182	2.2	193	42	1.9	3.5	11	816	0.13	88.3	68.9	4.4
		5	**787**	808	182	2.2	191	42	1.9	3.5	11	810	0.13	88.5	69.4	4.9
		6	**811**	825	182	2.2	191	41	1.9	3.5	11	835	0.14	90.1	73.7	5.9
		7	**848**	843	182	2.2	189	41	1.9	3.5	11	857	0.14	95.1	77	6.5
	Series III	8	**886**	871	223	2.0	**378**	61	2.6	3.1	10	831	0.16	93	81.31	4.6
																
PFR	Series IV	9	**799**	779	**126**	2.9	173	71	1.0	3.3	14.4	801	0.12	82.6	51	2.9
		10	**836**	813	**126**	2.9	166	68	1.0	3.3	14.4	841	0.13	88.5	67.7	2.4
	Series V	11	**875**	834	**340**	2.9	163	25	2.4	3.3	14.4	867	**0.13**	91.7	76.7	2.1
		12	**866**	837	**340**	2.9	176	27	2.4	3.3	14.4	866	**0.35**	90.5	73.4	2.9
		13	**866**	834	**340**	2.9	154	23	2.4	3.3	14.4	864	**0.41**	90.7	73.9	3.4
		14	**886**	828	**340**	2.9	153	23	2.4	3.3	14.4	861	**0.41**	92	77.6	3.9
																
Olive stone	Series VI	15	**779**	718	182	1.8	253	43	**2.4**	6.1	8.6	798	0.33	73.3	28.2	9.7
		16	**804**	763	182	1.8	255	44	**2.4**	4.8	8.6	798	0.33	77.4	39.3	10.2
		17	**805**	721	182	1.8	258	44	**2.4**	6.1	8.6	799	0.33	75.6	34.4	9.8
		18	**880**	768	222	1.8	259	36	**2.9**	4.8	8.6	844	0.34	82.8	53.7	6.9
		19	**887**	770	194	1.8	259	41	**2.5**	4.8	8.6	849	0.35	82.2	52.2	7.5

a Bold-faced figures indicate the operating parameter that was varied in the corresponding series.

**Table 5:** Operating Conditions and Main Results in the CLOU Unit during Taldinsky MVB Coal Combustion^a^

Fuel	Series	Test	*T*_FR_ (°C)	*T*_AR_ (°C)	*ṁ*_OC_ (kg/h)	*ṁ*_SF_ (kg/h)	*m*_FR_ (kg/MW_th_)	τ_OC_ (s)	ϕ (-)	λ (-)	Power (kW_th_)	*T*_CS_ (°C)	*u*_g,in cs_ (m/s)	η_CC_ (%)	*X*_C_ (%)	Ω_OD_ (%)
Taldinsky coal	Series VII	1	851	686	228	2.2	**205**	53	1.5	3.8	16.3	852	0.13	64.6	55.3	2.2
		2	836	768	228	2.2	**169**	44	1.5	3.8	16.3	852	0.13	44.9	30.4	2.1
		3	841	790	228	2.2	**147**	38	1.5	3.8	16.3	856	0.13	43.5	28.6	2
	Series VIII	4	837	804	228	2.2	141	36	1.5	3.8	16.3	855	**0.24**	38.4	22.1	2.9
		5	840	801	228	2.2	143	37	1.5	3.8	16.3	857	**0.35**	39.3	23.3	4
		6	831	801	228	2.2	135	35	1.5	3.8	16.3	851	**0.47**	38.7	22.5	5.2
		7	835	786	228	2.2	135	35	1.5	3.8	16.3	850	**0.47**	42.5	27.3	5.4
		8	846	775	228	2.2	135	35	1.5	3.8	16.3	847	**0.46**	44	29.3	5
		9	847	785	228	2.2	142	37	1.5	3.8	16.3	853	**0.47**	44.9	30.5	4.5
		10	841	759	228	2.2	133	34	1.5	3.8	16.3	841	**0.55**	45.9	31.7	6.3
		11	846	768	228	2.2	133	34	1.5	3.8	16.3	848	**0.55**	48	34.4	5.9
	Series IX	12	852	883		3.4	109		0.7	2.5	**25.1**	849	0.35	30.9	12.7	6.5
		13	862	963		5.2	65		0.5	1.6	**38.4**	862	0.35	33.4	15.9	5.5
		14	859	999		6.8	48		0.4	1.2	**50.2**	857	0.35	40.7	25.2	5.2
		15	860	1033		6.8	47		0.4	1.2	**50.2**	858	0.35	39.7	23.9	5.7
		16	864	1063		6.8	47		0.4	1.3	**50.2**	856	0.35	34.8	17.6	5.8

a Bold-faced figures indicate the operating parameter that was varied in the corresponding series.

In total, 35 steady state conditions were accomplished. In all the series of experiments, air was used as the fluidizing medium and reoxidizing agent in the air reactor with a flow rate between 30000 and 57000 l_N_/h. In the fuel reactor, the gas flow rate introduced oscillated between 8000 and 10000 l_N_/h. In the fuel reactor and carbon stripper, carbon dioxide, water, nitrogen, or a combination of them were used as fluidizing/gasifying agents. The circulation flow rate was measured through the diverting solid valve located below the air reactor cyclone.

Table 4 shows the experimental conditions during combustion of the three types of biomass. Series I–III used pine sawdust as fuel. Series I includes the analysis of the influence of the excess air ratio, λ, during pine sawdust combustion. The excess air ratio, λ, correlates the oxygen fed in the air reactor with the amount needed to reach full combustion of the fuel. It was calculated according to the following equation:

$$\lambda =\frac{\mathrm{oxygen}\mathrm{fed}\mathrm{in}\mathrm{the}\mathrm{air}\mathrm{reactor}}{\mathrm{oxygen}\mathrm{demanded}\mathrm{for}\mathrm{the}\mathrm{complete}\mathrm{combustion}\mathrm{of}\mathrm{the}\mathrm{fuel}}$$ 1

Series II analyzes the effect of the fuel reactor temperature on the key indicator parameters. In addition, Series III is constituted by test 8, obtained under experimental conditions selected to optimize the performance with pine sawdust.

Series IV and V investigated the effect of solid circulation rate and the gas velocity at the inlet of the carbon striper (CS) while PFR was used as a fuel. The solids circulation rate is linked to the oxygen carrier to fuel ratio, ϕ, which indicates the available oxygen in the circulating oxygen carrier. It is calculated as the amount of oxygen transported from the air reactor to fuel reactor by the oxygen carrier divided to the required amount of oxygen for full combustion of the fuel:

$$\phi =\frac{\mathrm{potential}\mathrm{oxygen}\mathrm{available}\mathrm{in}\mathrm{the}\mathrm{oxygen}\mathrm{carrier}}{\mathrm{oxygen}\mathrm{demanded}\mathrm{for}\mathrm{the}\mathrm{complete}\mathrm{combustion}\mathrm{of}\mathrm{the}\mathrm{fuel}}$$ 2

Finally, Series VI also evaluated the effect of the fuel reactor temperature during the combustion of olive stones.

Table 5 illustrates the experimental conditions tested in the combustion experiments with Taldinsky MVB coal. Series VI, VII, and VIII maintained the fuel reactor temperature, and the effects of the solids inventory in the fuel reactor, the gas velocity at the inlet of the carbon stripper, and the increase of the thermal power up to 50 kW_th_, respectively, were evaluated.

### 2.5 Data Evaluation

Performance of the CLOU process was assessed through different parameters. First, the mass balances for C and O were accomplished. This allowed us to check the solid fuel conversion. Note that nonconverted carbon in the CLC unit is assumed to be elutriated from the FR as unconverted char.

Three key performance indicators (KPIs) considered. CO_2_ capture efficiency, η_cc_, was defined as the proportion of carbon exiting from the fuel reactor relative to the total carbon in the fuel. It is usually considered that all carbon in gases from the FR is captured in the form of CO_2_, CO, CH_4_, or other hydrocarbons, since the use of an oxygen-polishing step downstream from the FR is proposed to achieve complete oxidation to CO_2_ and H_2_O of the compounds that have not been converted into the FR.

$$\eta_{\mathrm{CC}}=\frac{\mathrm{carbon from fuel}\mathrm{in}\mathrm{gases from fuel reactor}}{\mathrm{carbon in fuel fed}}$$ 3

CO_2_ capture efficiency is dependent on char conversion, which is defined as follows:

$$X_{\mathrm{char}}=\frac{\mathrm{carbon from}\mathrm{char}\mathrm{converted}\mathrm{in}\mathrm{the}\mathrm{FR}}{\mathrm{fixed}\mathrm{carbon}\mathrm{in}\mathrm{the}\mathrm{fuel fed}}$$ 4

Ω_OD_ is defined as the total oxygen demand of the CLC process, which is related to the oxygen required to burn the unconverted gases at the FR outlet in the oxygen polishing step located after this reactor.

$$\Omega_{\mathrm{OD}}=\frac{\mathrm{oxygen demaned}\mathrm{for}\mathrm{the}\mathrm{complete combustion}\mathrm{of}\mathrm{gases from FR}}{\mathrm{oxygen demanded}\mathrm{for}\mathrm{the}\mathrm{complete combustion}\mathrm{of}\mathrm{the}\mathrm{fuel}}$$ 5

## 3 Results and Discussion

### 3.1 Results for Different Types of Biomass

The operation of the 50 kW_th_ unit during the experimental campaign presented in this work was smooth. No notable operational problems were related to fluidization of the material. As an example, the concentrations (dry basis) at the outlet of fuel and air reactors are shown for Series VI in Table 4 (olive stone) and in Figure 3.

**Figure 3:**
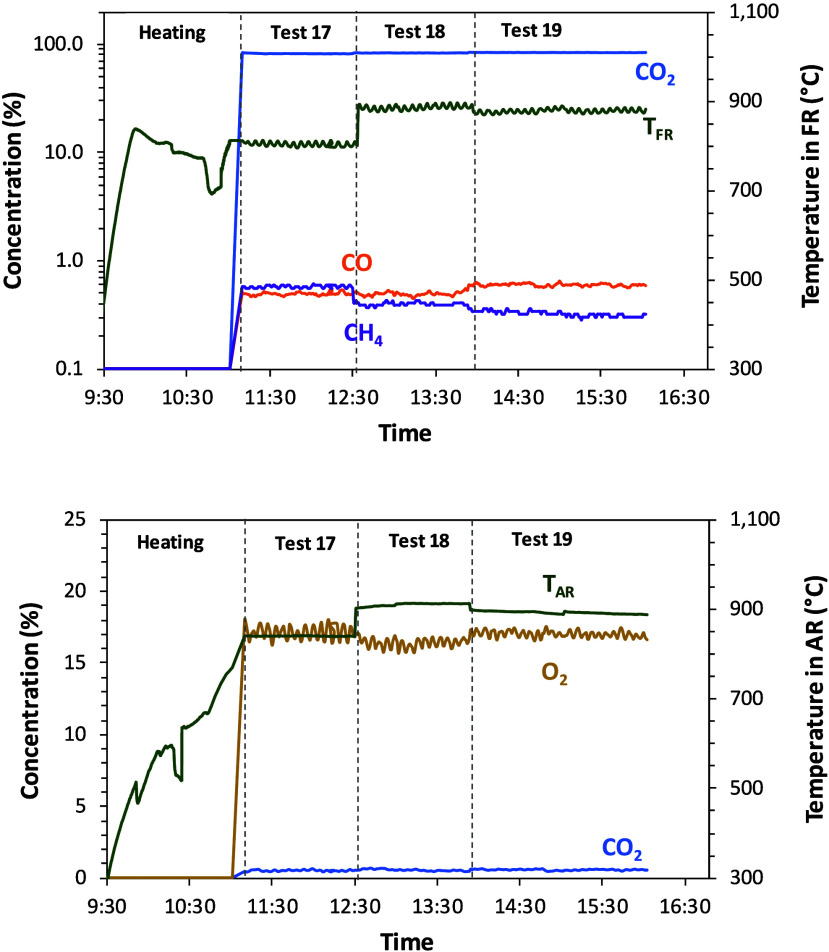
Example of fuel and air reactor outlet concentrations (dry basis) Series VI in Table 4 (olive stone).

The evaluation of the results in the 50 kW_th_ CLC unit corresponding to biomass is presented first. Results in Table 4 were grouped by the type of biomass burned. However, the evaluation of the results obtained are focused not only on the type of fuel used but also on the effect of different operating parameters on the key performance indicators (KPIs), which can be observed regardless of the fuel used. It is already known that CO_2_ capture efficiency also depends on the char conversion rate, and both are affected by the temperature in the fuel reactor and the residence time of the oxygen carrier particles in the fuel reactor. Thus, both KPIs (CO_2_ capture efficiency and *X*_char_) are included together in many of the figures presented through this work. The total oxygen demand is affected by several operating parameters, such as the fuel reactor temperature, solids inventory in the fuel reactor, the solids circulation rate/oxygen-carrier-to fuel ratio (ϕ), and the value of λ.

### 3.2 Excess Air Ratio (λ)

Previous studies with CuMn-mixed oxides already reported that this variable may affect the performance of the fuel reactor.^13^ Low values of λ are translated into a low oxygen concentration in the air reactor, and due to this reason, the regeneration of the oxygen carrier may not proceed completely. The lack of regeneration of the oxygen carrier influences the oxygen release rate in the fuel reactor which becomes lower, thus reducing char conversion and, in turn, CO_2_ capture efficiency. Series I with pine sawdust was used to assess the effect of the excess air ratio (λ) on the KPIs. Figure 4A,B exemplifies this behavior in the experiments with pine sawdust and Cu30MnFe oxygen carrier.

**Figure 4:**
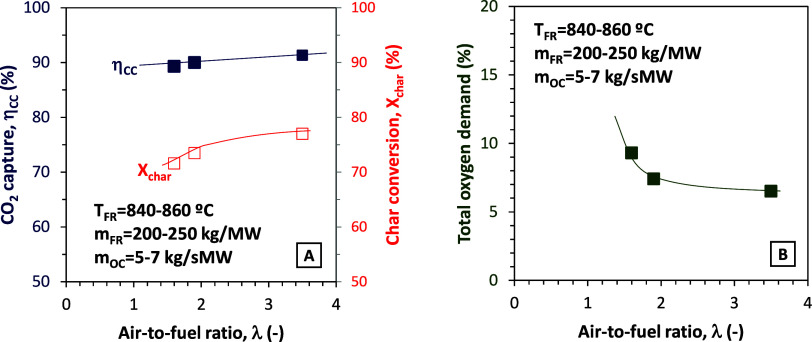
Effect of excess air ratio on (A) CO_2_ capture efficiency and char conversion and (B) total oxygen demand (fuel, pine sawdust).

According to the figures, the variation in the λ parameter form 1.6 to 3.5 represented a reduction in the total oxygen demand from 9.3 to 6.5%. Therefore, and in order to obtain reliable results in the experimental campaign, a value of λ close to 3.5 was maintained in the rest of the experiments.

### 3.3 Fuel Reactor Temperature (*T*_FR_)

The fuel reactor temperature is one of the main operating parameters influencing the performance of the CLOU process. It should be noted that the fuel reactor temperatures reached in the experiments in the present work were low when compared with previous studies with the same oxygen carrier but in a lower-scale unit (1.5 kW_th_).^14^ In the present study, temperatures did not exceed 850–860 °C (except a few tests). Another factor to be considered in the experiments in the 50 kW_th_ unit when compared to those at 1.5 kW_th_ is the fluidization agent used in the fuel reactor. In the present study, H_2_O or mixtures CO_2_/H_2_O have been used together with nitrogen as the fluidizing agents in the fuel reactor, while in the 1.5 kW_th_ unit the fuel reactor bed was fluidized exclusively with nitrogen. Thus, in the experiments in the 50 kW_th_ unit, char might be consumed by not only the oxygen released by the oxygen carrier but also gasification with H_2_O/CO_2_. At the temperatures in the present work, both mechanisms may contribute to the final value of char conversion.

Figure 5A,B jointly summarizes the results obtained on the effect of the fuel reactor temperature on the KPIs for all types of biomass used as well as Taldinsky coal. In the comparison, the experimental data shown correspond to Series II (pine sawdust), test 11 (PFR), Series VI (olive stone), and test 3 (Taldinsky coal). In any case, the increase in the fuel reactor temperature favors char conversion. However, the difference between biomass and coal is clear, since higher char conversion and CO_2_ capture efficiencies were reached by the three types of biomass when compared to coal at similar fuel reactor temperatures.

**Figure 5:**
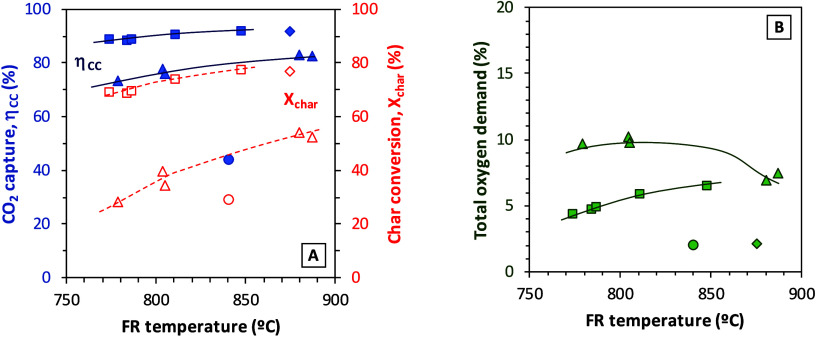
Effect of the fuel reactor temperature on (A) CO_2_ capture efficiency and char conversion and (B) total oxygen demand for different types of fuel. Experimental conditions: solids inventory in the FR, 150–250 kg/MW_th_; solids circulation rate, 4–6 kg/(s·MW); solids residence time in FR, 25–40 s; materials, (blue filled squares) pine sawdust, (blue filled diamonds) pine forest residue (PFR), (blue filled triangles) olive stone, and (blue filled circles) Taldinsky coal.

The effect of the fuel reactor temperature on the total oxygen demand in Figure 5B should be analyzed in more detail. In the case of pine sawdust (Series II), the total oxygen demand increases with the fuel reactor temperature. The increase correlates with the increase in char conversion, meaning that, as more char is converted, more gaseous gasification products are generated and not all of them burned. Thus, an increase in the unburned compounds at the fuel reactor outlet can be observed. In order to further increase the CO_2_ capture efficiency and decrease the total oxygen demand of pine sawdust, higher temperatures in the fuel reactor were combined with an increase in the solids inventory in the fuel reactor in Series III (test 8) in Table 4. As can be seen in Figure 6, test 8 confirmed the improvement in CO_2_ capture efficiency (93%) and total oxygen demand (4.6%) when the fuel reactor temperature and solids inventory in the fuel reactor were optimized.

**Figure 6:**
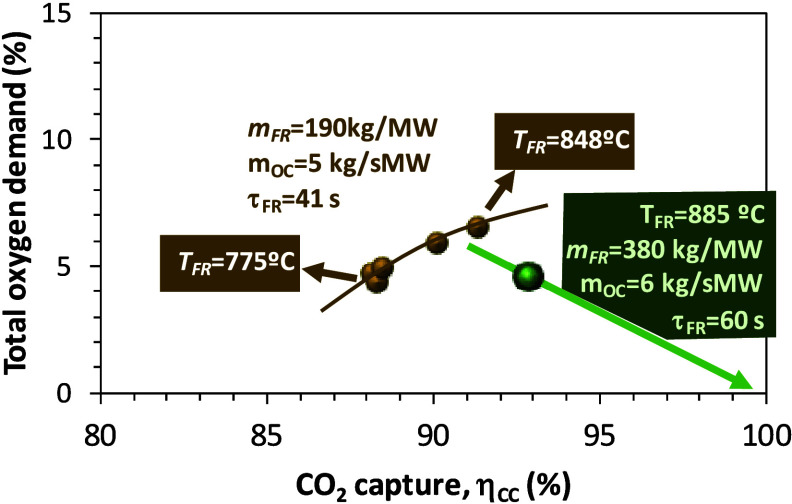
CO_2_ capture efficiency and total oxygen demand for Series II and III (fuel, pine sawdust).

In the experiments with olive stone (Series VI) in Figure 5B the trend seems to be the opposite to that of pine sawdust. The total oxygen demand decreases as the fuel reactor temperature is higher. In this case, a joint effect of the increase in the fuel reactor temperature with an increase in the solids circulation rate in tests 18 and 19 may justify the decrease in the oxygen demand. The increase in temperature may favor gasification and gas–solid reactions. Moreover, when the solids circulation increases, the average reactivity in the fuel reactor bed increases, although at high solids circulation rates, changes in the average reactivity are small.^27^ It should also be highlighted that the highest total oxygen demand values for all of the fuels were those corresponding to olive stone tests. It seems that reactivity of the char from olive stones is lower, as it has been already observed in experiments in the 1.5 kW_th_ unit, operating under similar conditions.^14^ On the contrary, the lowest total oxygen demand was reached in tests with pine forest residue (PFR) and Taldinsky coal (about 2%).

### 3.4 Solids Circulation Rate and Gas Velocity in the Carbon Stripper

Experimental data obtained with PFR in Series IV and V further analyze the joint effect of fuel reactor temperature and solids circulation rate in Figure 7. Results in Figure 7A were obtained at different temperatures and solid circulation rates; thus, the residence time in the fuel reactor of the particles was affected. However, following the trends outlined by the data in the figure, it may be possible to obtain higher values of char conversion and CO_2_ capture efficiencies for a determined fuel reactor temperature when the solid circulation rate was lower, since the residence time of the particles in the fuel reactor increased. The total oxygen demand value, in Figure 7B is also affected by the solid circulation rate. As it has been previously observed before in the experiments with olive stone in Series VI, the total oxygen demand decreased when the solids circulation rate was higher. Moreover, the experiments with PFR plotted in Figure 7B also revealed another parameter influencing the total oxygen demand, which is the gas velocity in the carbon stripper (CS). Open symbols in Figure 7B show the total oxygen demand values obtained for different gas velocities in the CS at the highest solid circulation rate (6.5 kg/sMW) (Series V). Higher values of total oxygen demand were reached with the highest gas velocity in the carbon stripper.

**Figure 7:**
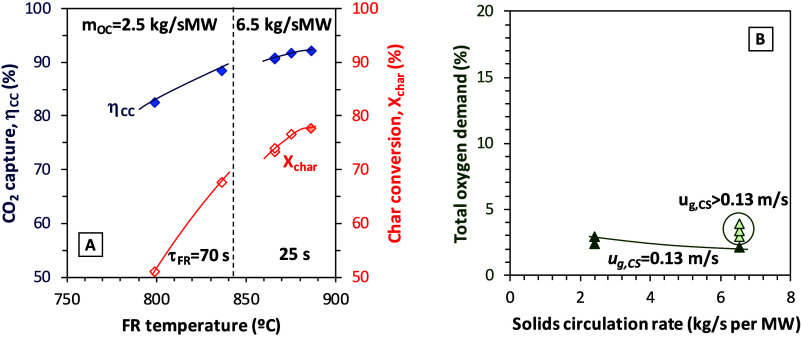
Effect of fuel reactor temperature and solids circulation rate on (A) CO_2_ capture efficiency and char conversion and (B) total oxygen demand (fuel, pine forest residue, PFR).

Figure 8A analyzes this behavior in greater depth. In this figure the total oxygen demand increased with the increase in the gas velocity in the carbon stripper. However, this does not translate into an improvement in char conversion, since it decreased as the gas velocity in the carbon stripper is higher. In this case with PFR, the temperature in the FR seems to have a more relevant effect on the total oxygen demand than the gas velocity in the CS.

**Figure 8:**
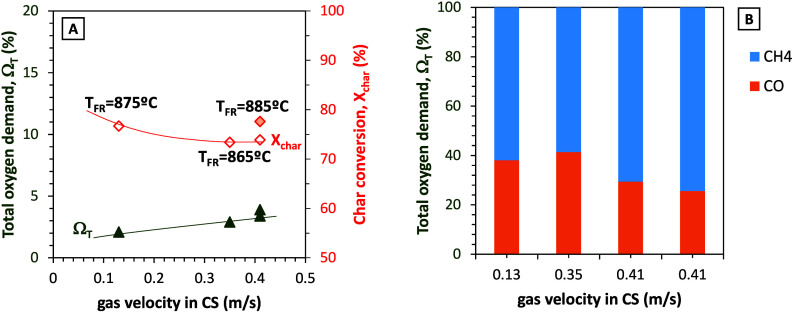
(A) Effect of gas velocity in the carbon stripper on char conversion and total oxygen demand. (B) Contribution (%) to the total oxygen demand value of the main unburned products (fuel, pine forest residue, PFR).

The contribution to the total oxygen demand of the main unburned products was evaluated for the tests in series V and is represented in Figure 8B. It should be specified that, in any of the experiments with biomass, hydrogen was detected as unburned gas. In the figure, the contribution of methane to the final total oxygen demand value increases as the gas velocity in the carbon stripper increases, indicating that volatiles (with CH_4_ as a representative species) are worse burned when the velocity in the carbon stripper is higher. As a consequence, it may be considered whether the option of incorporating a carbon stripper into the CLC unit is appropriate when biomass fuels are used, since in the case of PFR it has been proven that it contributes to the increase in total oxygen demand.

Finally, Figure 9 presents a general overview based on selected data for the different types of fuels considered in the present work. Total oxygen demand values are plotted against the CO_2_ capture efficiencies for the three types of biomass and Taldinsky coal. All of the experiments were performed in the 780–880 °C range for temperature in the fuel reactor, but it should be highlighted that the experiments with biomass were done at the highest temperatures within the range mentioned. Under the conditions in the figure, the values of total oxygen demand were in any case below 10%. As mentioned, olive stones presented the highest oxygen demand values followed by pine sawdust, while coal and PFR pellets presented the lowest values. In terms of CO_2_ capture efficiency, the largest values correspond to pine sawdust and PFR. Therefore, one of the conclusions that can be obtained from this comparison is that burning biomass under the CLOU process at TRL 5 brings combustion results closer to the hypothetical goal of achieving zero oxygen demand and 100% capture efficiency than in previous experiments working in the same unit with the same types of biomass but using an oxygen carrier without CLOU properties.^26^ Another conclusion is the potential advantage of using fuel pellets as feedstock for the 50 kW_th_ CLOU unit instead of particulated fuel. The pellets allow for slow devolatilization which facilitates volatile combustion by the oxygen carrier. Moreover, the larger size of the pellets compared to particles of fuel avoids the tendency to be entrained, thus favoring char conversion and CO_2_ capture efficiency.

**Figure 9:**
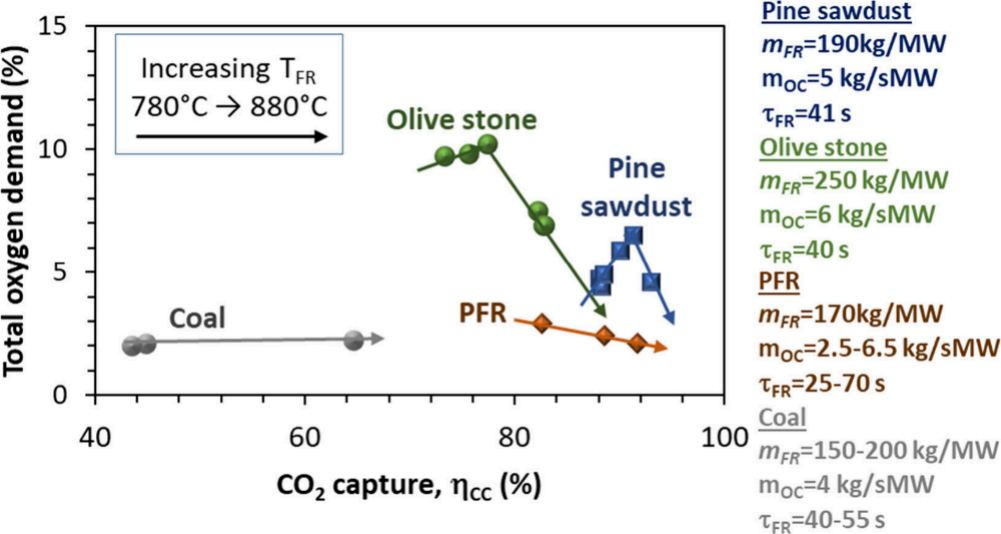
CO_2_ capture efficiency and total oxygen demand for different fuels used with Cu30MnFe in the 50 kW_th_ unit (*T*_FR_ = 780–880 °C).

Further analysis of the most optimized tests in each series with the different types of biomass in Figure 10 indicated that the gas mainly contributing to the oxygen demand is methane. This means that volatile conversion in the fuel reactor should be improved in order to further decrease the total oxygen demand value.

**Figure 10:**
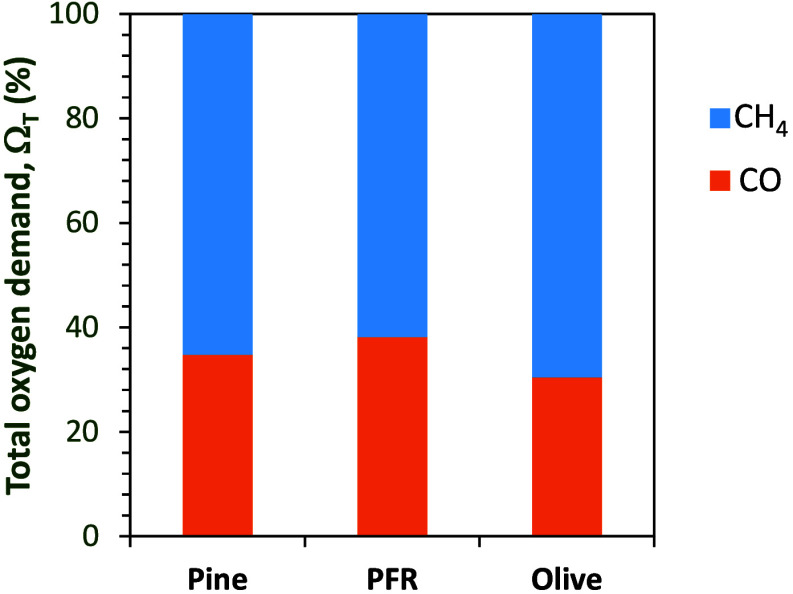
Contribution (%) to the total oxygen demand value of the main unburned products for optimized tests with the three types of biomass.

### 3.5 Results for Taldinsky Coal

Most of the experiments with Taldinsky coal were performed at temperatures close to 850 °C. The experiments with this sub-bituminous coal tried to analyze the effect of other operating conditions affecting the performance of the CLOU unit.

### 3.6 Solids Inventory in the Fuel Reactor

Series VII with Taldinsky coal investigated the effect of the solids inventory in the fuel reactor. Figure 11 presents the results obtained for three different values of this parameter.

**Figure 11:**
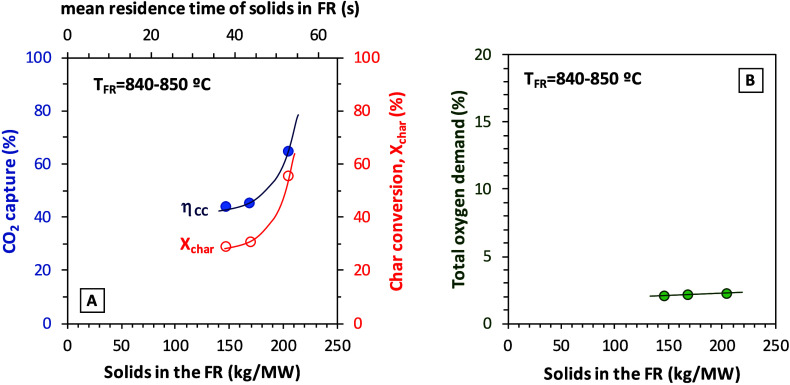
Effect of the solids inventory in the fuel reactor on (A) CO_2_ capture efficiency and char conversion and (B) total oxygen demand (fuel, Taldinsky coal).

As can be seen in Figure 11A with data from Series VII, char conversion and thus CO_2_ capture efficiency are significantly improved by increasing the solids inventory in the fuel reactor. Higher solids inventories are linked to a higher residence time of solids, which favors char conversion. However, the oxygen demand barely changed. This may indicate a trade-off between the effect of the increase in char conversion and the higher oxygen availability due to the increase in the solids inventory.

### 3.7 Gas Velocity in the Carbon Stripper

Series VIII was dedicated to the evaluation to the effect of the gas velocity in the carbon stripper, in this case, while the temperature in the fuel reactor was approximately constant. The evaluation of the effect of the changes in this parameter has been previously attempted in the experiments with PFR. However, results obtained with coal differ from those obtained with PFR as, explained next.

In Figure 12A, the increase in the gas velocity in the carbon stripper was translated into an increase in the char conversion and, thus, in the CO_2_ capture efficiency. The CO_2_ capture increased from ca. 39% at low *u*_CS_ values (0.2–0.3 m/s) to ca. 47% when *u*_CS_ increases to 0.55 m/s. This represents an increase of 20% in the CO_2_ capture, which is similar to what was obtained in a previous work with coal and ilmenite as the oxygen carrier.^24^ This larger effect of the carbon stripper highlights another of the results in Figure 12A, which is the lower reactivity of Taldinsky coal compared to biomass, a result already observed in numerous previous studies, including studies with this same Cu30MnFe oxygen carrier in a 1.5 kW_th_ CLC unit.^14^ At 850 °C, the char conversion values were in the 30–50% range and had CO_2_ capture efficiencies around 50%, while, in the case of biomass, even for the less reactive olive stones, char conversion values were 40–60% and the CO_2_ capture efficiencies were between 75 and 85%. Thus, improved CO_2_ capture efficiencies and char conversion could be expected if the temperature for coal combustion could be increased to more common combustion temperatures (∼950 °C).

**Figure 12:**
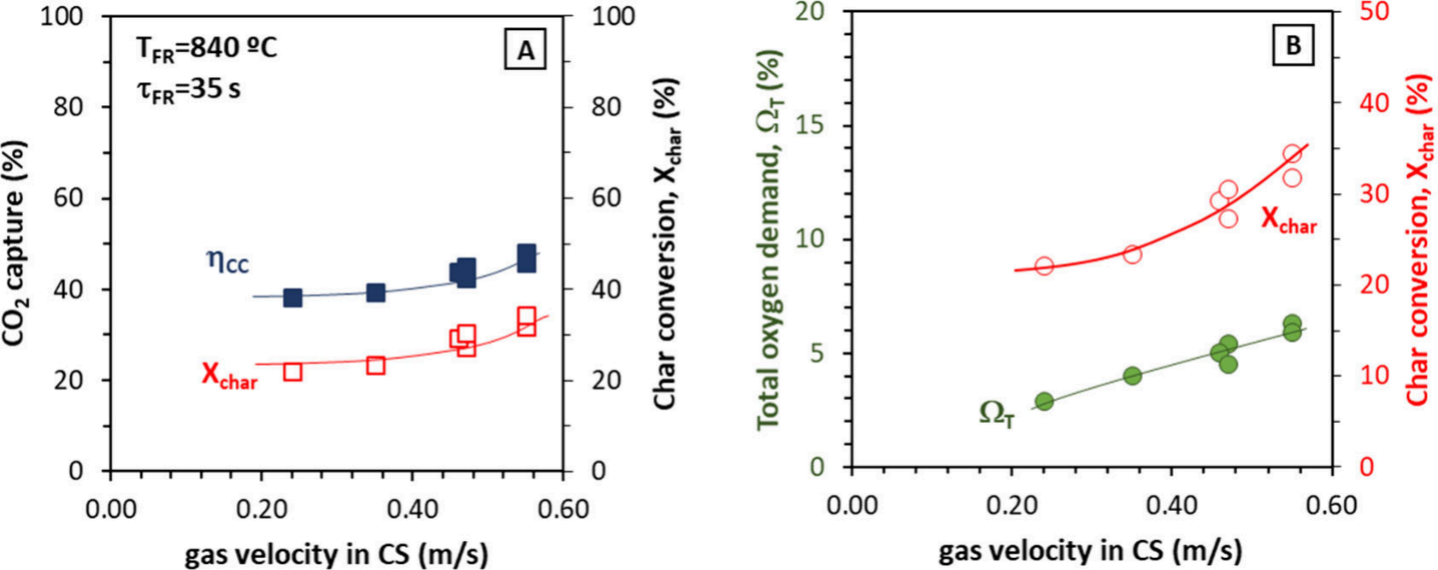
Effect of gas velocity in the carbon stripper on (A) CO_2_ capture efficiency and char conversion and (B) total oxygen demand (fuel, Taldinsky coal).

In Figure 12B, the total oxygen demand also increased with a higher gas velocity at the carbon stripper. A higher gas velocity in the carbon stripper enhanced char conversion, and therefore, more gases were released in the fuel reactor, which would explain the increase in the total oxygen demand. This behavior is opposite to what was observed in Figure 8A in the experiments with PFR where the effect of the fuel reactor temperature on the char conversion was more relevant than the changes in the gas velocity in the carbon stripper. These different effects of the increase in the gas velocity of the CS can be explained by the analysis of the solid distribution in the FR circulating fluidized bed during the experiments with both types of fuel. Figure 13 shows the pressure drop in the riser of the fuel reactor of the 50 kW_th_ CLOU unit during the corresponding experiments with PFR and Taldinsky coal. It should be mentioned that the riser of the FR is where the combustion of volatiles takes place.

**Figure 13:**
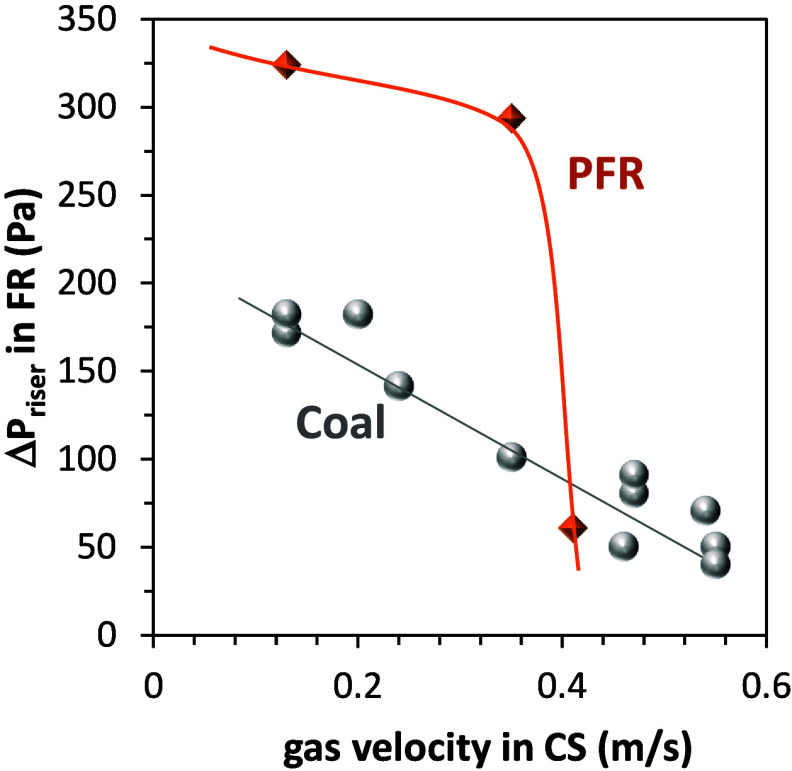
Pressure drop in the fuel reactor riser in experiments with different gas velocities in the carbon stripper using PFR and Taldinsky coal as fuel.

When there is a higher gas–solid velocity in the carbon stripper, the pressure drop in the fuel reactor riser decreases, indicating that fewer solids are present in the riser of the fuel reactor. This decrease is especially dramatic in the case of the experiments with PFR. Since PFR has a higher volatile content when compared to Taldinsky coal, it can be expected that the increase in the velocity of the carbon stripper would make more difficult the volatile combustion, and therefore an increase in the total oxygen demand could be observed although it did not correspond to an increase in char conversion.

### 3.8 Fuel Feeding Rate

Finally, Series IX with Taldinsky coal was accomplished with the aim of stepwise increasing the fuel load to the continuous unit up to 50 kW_th_. The temperature in the fuel reactor was set to 850–860 °C. However, during these experiments, it was not possible to measure the solids circulation rate in the unit, and therefore, the solids residence time cannot be calculated with accuracy. Nevertheless, very low values of residence time of the particles are suspected (∼10 s), since the behavior of the unit as the power was gradually increased suggests that the circulation of solids was extremely high. Considering these limitations, Figure 14 shows the evolution of the KPIs as the power fed to the unit increases.

**Figure 14:**
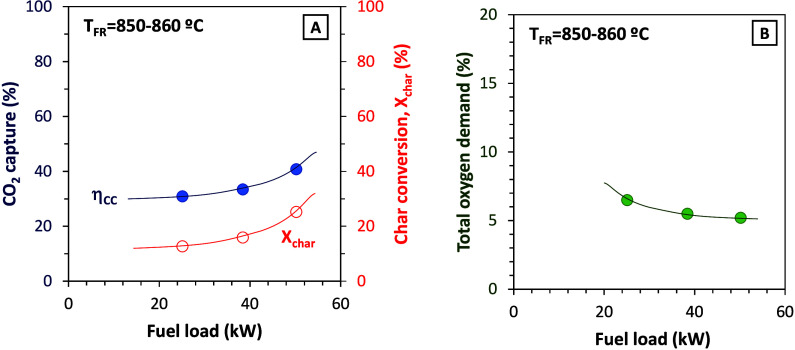
Effect of fuel load on (A) CO_2_ capture efficiency and char conversion and (B) total oxygen demand (fuel, Taldinsky coal).

According to the results in the figure, the increase in thermal power increased char conversion and CO_2_ capture efficiency as well as decreased the total oxygen demand. Thus, the increase in the fuel feeding rate improved the performance of the 50 kW_th_ CLOU unit. Nevertheless, it should be noted that at the end of this series of experiments and working with a fuel feed of 50 kW_th_ the temperature in the air reactor started to increase up to values higher than 1000 °C (see Figure 15).

**Figure 15:**
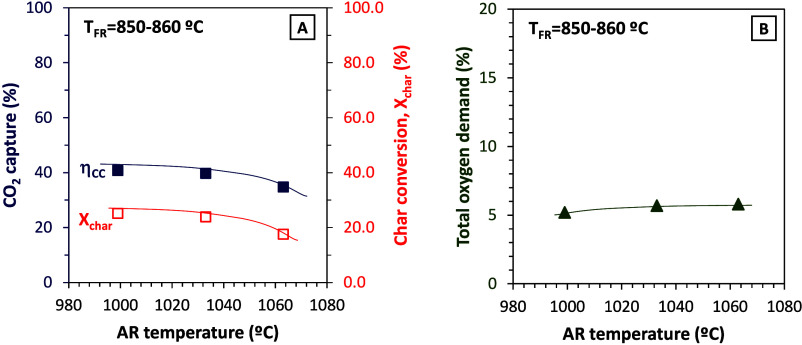
Effect of temperature in the air reactor on (A) CO_2_ capture efficiency and char conversion and (B) total oxygen demand (fuel, Taldinsky coal; tests at 50 kW_th_).

The increase in the air reactor temperature would cause difficulty in the proper oxygen carrier regeneration to CuO, thus limiting the CLOU effect in the fuel reactor. This would explain the decrease of char conversion and CO_2_ capture efficiency in Figure 15. Actually, the AR furnace should be switched off to avoid further increase in the AR temperature. The total oxygen demand did not vary much and was maintained around 5% in all of the experiments. Note that the specific solids inventory in the FR decreased from 150 to 47 kg/MW_th_ as the fuel load was increased. Therefore, the amount of solids in the CLC unit was enough to maintain the oxygen demand at low values, although the solids inventory was quite low. The good behavior of the CLOU process, even in the case of having low specific solids inventory, was yet observed at the 1.5 kW_th_ scale.^10^ As a conclusion from the tests in Series IX with Taldinsky coal, it can be said that CO_2_ capture efficiency and total oxygen demand can be maintained while increasing the fuel load up to the nominal power of 50 kW_th_. However, operating conditions should be significantly optimized in future tests, mainly by increasing the fuel reactor temperature above 900 °C, where the CLOU effect starts to be relevant and contributes significantly to combustion.

In the present experimental campaign, the conditions were so extreme in terms of the need of oxygen transfer that the oxygen carrier did not resist and broke, generating a large amount of fines. In future works, an exhaustive characterization of the evolution of the properties of the oxygen carrier according to the operating conditions will be presented. As future work also remains, the development of durable oxygen carrier particles for CLOU.

## 4 Conclusions

This work presents the results of the combustion of different types of biomass and a bituminous coal using an oxygen carrier with oxygen uncoupling capability and magnetic properties in a CLC unit composed of two interconnected circulating fluidized bed reactors at 50 kW_th_ (TRL 5). These results are relevant for the future scale-up of the CLOU technology.

In general, excess air ratios above 2 may be recommended in order to maximize the combustion efficiency. In the combustion of the different types of biomass, high capture efficiency values were obtained, up to 93% at a temperature close to 890 °C, with the reference biomass pine sawdust. However, complete combustion of the devolatilization/gasification products was not possible and oxygen demand values of 4.6% were obtained once the conditions were optimized with the same pine sawdust. The CO_2_ capture and oxygen demand values depended on the type of biomass, but the total oxygen demand did not exceed 10%. The results with biomass showed that the presence of a carbon stripper was not a condition for improving char conversion and, therefore, CO_2_ capture efficiency.

In the experiments with bituminous coal, the operating conditions could not be optimized to achieve high CO_2_ capture efficiencies, which were lower than those with biomass. However, the effectiveness of the carbon stripper in increasing the CO_2_ capture efficiency was demonstrated. The tests carried out with a high power of fed coal showed that it is possible to achieve low total oxygen demands on coal combustion (of the order of 5%) even at temperatures below 900 °C.

During the experimental campaign, the oxygen carrier particles suffered severe deterioration of their mechanical properties. In future works, the formulation of the oxygen carrier should be modified in order to obtain particles with the desired high lifetime during its use in a CLOU unit.
